# Abstracts and Gleanings

**Published:** 1879-09-20

**Authors:** 


### ABSTRACTS AND GLEANINGS.

**Value of the Hypophosphites.**—In the treatment of pulmonary consumption, we do not administer the hypophosphites, or any one of them, by routine, or indiscriminately, or in any case rely on them solely. And just here permit me to say that it is astonishing that physicians should so frequently order such therapeutically incompatible combinations. Often we see the hypophosphites of lime and potash in the same prescription, and it may be for a patient far advanced in consumption, when every tyro in medicine is familiar with the fact that the potash salts, as a rule, have a deliquescing effect upon the vital tissues, increasing the congestion and augmenting the discharge from mucous membranes. And yet, here, in a case where an astringent and roborant effect is plainly called for, physicians professing to be scientific prescribe a remedy that, if it produce its characteristic well known effect, will only hurry the patient to the grave, and withal make his journey more miserable as well as sadly brief. In answer to our inquiries, manufacturing pharmaceutists tell us that the reason why they continue to prepare such therapeutically incompatible mixtures of the hypophosphites as now flood our markets, is because physicians will have them thus. While it is a well known and fully established fact, that there are no two of the hypophosphites but that have each its characteristic and peculiar therapeutic properties. And while it is a familiar fact that medicines of more or less similar or dissimilar therapeutic properties may be combined, to the advantage of the patient, yet it seems to us impossible that the hypophosphite of potash, in any combination or quantity, should benefit a consumptive patient whose lungs were more or less rapidly softening and breaking down; yet it is prescribed for such, certainly to their injury.

Not thinking it necessary to consume time and space with the minute details of special cases to illustrate our mode of administering the hypophosphites in the treatment of pulmonary consumption, or to endeavor to substantiate the fact of their usefulness in that most fatal disease, we deem it sufficient to give a mere general outline of our usual plan. And in doing so we do not presume to tell anything new or original as to their use, but beg permission to refer you to our teachers—Thorowgood, Van Holsbeck, Bougard, Berchon, Dickson, Churchill, Campbell and others, who have written so well upon the subject; and though they are, or were, physicians acknowledged to be of the first ability and highest standing in their respective countries, yet their teachings have not been so fortunate in catching the popular breeze as those of the cod liver oil advocate.

We have used, in the treatment of pulmonary consumption, the hypophosphites of soda, lime and quinine. We find the soda salt most frequently applicable, next that of quinine, while in some cases, where an astringent and roborant effect is indicated, that of lime serves an invaluable purpose. The hypophosphites of soda and lime we administer in simple syrup solution, and that of quinine usually in pills. In their administration we follow the general instructions as laid down by Dr. J. F. Churchill, in his late work on this subject, and to which we beg permission to refer you for much that we would repeat and endorse,

2

as substantiated by our own experience and observation. We have used the hypophosphites, as manufactured by Swann, of Paris, to some extent, but usually we employ those of American manufacture, by Powers and Wightman, and Rosengarten & Son, and have not observed any appreciable difference between those of foreign and domestic manufacture. We usually administer the hypophosphites of soda and lime three to six grains, and that of quinine about half so much, three times a day. We often give the hypophosphite of quinine for one or two weeks before beginning with the alkaline hypophosphites. For most cases require more or less preliminary treatment, such as may be indicated in each case, before they are in suitable condition to begin a hypophosphite course. And those patients of hemorrhagic diathesis can profitably take much larger doses if two or three grains of ergotin be administered simultaneously three times a day. We have administered this amount of ergotin continuously for two to five months, or longer, and yet have never observed any bad effects follow, but on the other hand, the patient would steadily improve. We have never observed a single instance of any indication of dry gangrene following the use of ergotin, as mentioned in some old medical works. The old idea that dandelion is a specific, to a certain extent, for pulmonary consumption, is not without foundation; the beneficial results following its long continued use are probably owing to its effects upon the biliary organs, thus assisting in the assimilation of carbonaceous compounds.

For the arrest of hemoptysis, ipecac, in ten to twenty grain doses, is very efficient, and if taken in smaller non-emetic doses it is the best preventive we know of. Besides, it acts well in non-emetic doses on the digestive organs and upon the nervous system, especially the sympathetic branch of it. For the relief of night sweats, we have found the following very efficient:

℞ Oxidi zinci........................................ gr. j.

Acidi pyrogallici.................................. gr. ij.

Sulph. atropiæ...................................... gr. 1-100.

M. Ft. pil. j.

Dose, one to two pills one to three times a day.

To check consumptive diarrhœa, we generally use the following:

℞ Salicin............................................. gr. iij to vj.
Sub. nit. bismuth ................................ gr. vj to xij.

Dose, one such powder every three to six hours, or as occasion may require.

The hypophosphite of quinine we often administer in the following combination, under the name of Compound Pulmonary Alterative Pills:

℞ Hypophosphite quinine.......................... gr. 1.
Iodide of arsenic.................................. gr. 1-16.
Sulphide of calcium............................... gr. 1.
Ext. nux vomica.................................... gr. ⅛.
M. Ft pil.

Dose, one pill three times a day.

We have these pills, as well as many other kinds, made to order in New York or Philadelphia, and sugar or gelatine coated, in large quantities at a time.

Nux vomica and lactopeptine will often be found to render valuable service as aids to digestion.—*Pacific Medical Journal.*

**Treatment of Nocturnal Seminal Emissions.**—In all cases of frequent nocturnal emissions, the genital organs should be examined, and whether phimosis exists or not, if the prepuce be long and redundant, circumcision is to be recommended. A very marked varicocele may also render surgical interference desirable.

The hygienic rules to be given to the patient are very simple. It is better that the most substantial meal in the twenty-four hours should be taken at noon; the supper should be light, and food and drink be entirely avoided in the evening; the bed-chamber should be well ventilated, a hair mattress preferred to a feather bed, and much covering avoided. The patient should sleep upon his side, and not upon the back; a small pillow placed between the knees, so as to separate the thighs and prevent the scrotal organs from becoming heated, is sometimes desirable; and the patient should rise as soon as he wakes, emissions occurring most frequently during the semi-consciousness of the early morning nap.

Tobacco in every form should be prohibited, since it not only increases the general irritability of the nervous system, but appears to have a direct influence in diminishing the tone of the genital organs, and thus favoring seminal emissions.

Above all, as already stated, the mind of the patient should be distracted from his complaint by constant occupation, and his general health be promoted by a plain but nourishing diet, and by daily outdoor exercise, not carried to fatigue, since it is found by experience that when the strength is exhausted, an emission is more likely to occur.

Many of these patients also have constipated bowels, and means should be taken to secure a daily stool.

As a rule, no other measure than the above are required. It is to be understood, however, that any weakness and irritability of the nervous system may require the administration of tonics, a change of climate, etc. For this purpose I have found the two following prescriptions of good service :

℞ Ferri et quiniæ citrat........................................ ʒ iij.

Strychniæ sulph............................................... gr. j

Acidi phosphoric, dilut....................................... ℥ ss.

Syrup aurantii................................................ ℥ ij.

Aquam ad...................................................... ℥ iv. M

Dose, a teaspoonful in water, after each meal.

℞ Strychniæ sulph.............................................. gr. j.
Acidi phosph. dilut........................................... ℥ iij M

Dose, a teaspoonful three times a day, after eating.

The tincture of the chloride of iron, and also ergot, have been supposed, and I think justly so, to have a special tonic effect upon the genital organs; but they must be given in large doses, as, for instance, from half a drachm to a drachm of either the tincture of iron or the fluid extract of ergot (Squibb's), in water, after each meal. They may be combined, as in the following prescription :

℞ Tinc. ferri chloridi......................................... ℥ iij.

Fxt. ergotæ fl. (Squibb's).................................... ℥ iij. M

Dose, a teaspoonful in water, after each meal.

As a direct means of diminishing the frequency of the emissions, however, the following is often found to be most efficacious :

| | | |
|---|---|---|
| ℞ | Potassii bromidi | ℥ j. |
| | Tinc. ferri chloridi | ℥ j. |
| | Aquæ | ℥ iij. |

Dose, from one to two teaspoonfuls in water, after each meal and at bed-time.

Mention has already been made of the advisableness of circumcision when the prepuce is long. It may also be found, upon the introduction of a sound, that the urethra is over sensitive, especially in the prostatic region. In such cases the introduction of a cold sound of full size, at first every third or fourth day, and afterward with greater frequency, will generally afford relief to the hyperæsthesia. I sometimes inject into the prostatic urethra about ten drops of a solution of nitrate of silver of the strength of twenty grains to the ounce of water, by means of a deep urethral syringe, or Guyon's flexible catheter and syringe. The severe cauterization with the *porte-caustique* of Lallemand should by all means be avoided.—*American Practitioner.*

**Intravenous Injections.**—Dr. Griswold, in N. Y. Medical Record, says, that he has successfully employed a new method of ressuscitation. Among several cases we extract the following :

While I was obliged to admit that the case was hopeless, judged by ordinary standards, and beyond the reach of ordinary stimulants, I could not help feeling that heroic measures were specially indicated. The source of trouble—fluid compressing a lung and displacing the heart—had been removed; if the patient could be stimulated to breathe deeply and profit by its disappearance, there seemed to be good reason to hope for her recovery. Selecting a prominent superficial vein in the radial region, I exposed it by an incision through the skin. I then injected slowly into it a drachm of ammonia solution (equal quantity of aqua ammonia and water), taking care that the point of the hypodermic needle was free in the lumen of the vessel. This done, I placed my hand over the patient's heart and waited. In fifteen seconds, I felt a marked increase in the force of pulsation. In about two minutes there was a strong pulse of a hundred, which was plainly perceptible at the wrist. A minute later the patient sighed deeply; the color came back to her lips, her eyes moved and began to show signs of returning intelligence. On being urged, she swallowed without difficulty two ounces of strong egg-nog. After a few deep inspirations, she breathed more regularly and easily; her pulse was strong and tense, ranging between 100 and 110. Half an hour afterward she was perfectly conscious, and reported herself comfortable, though weak. Pulse 90, regular, strong. Respirations 26, easy and natural. Swallowed easily and willingly small quantities of egg-nog. During the afternoon and evening, patient continued to improve. Pulse 80—90 and strong; respirations 20—30 and easy. Patient passed a good night, sleeping most of of the time; was bright and refreshed in the morning.

May 7. Steady improvement since last note. Sat up for two hours to-day, and ate a lamb chop with relish.

May 17. Patient sits up nearly every day, and is gaining strength.

N. B.—Improvement has been uninterrupted since the injection of ammonia. No depression has been observed following the stimulant action of that remedy, nor has there occurred an unpleasant symptom which could be attributed to it.

The case described seems to satisfactorily establish :

1. That the intravenous injection of ammonia is a prompt and powerful means of stimulation, acting efficiently in cases where other measures are of no avail.

2. That no bad effects follow its employment.

While the importance of the above deduction is obvious as a matter of general therapeutic interest, they seem to have a special significance in connection with those operations whose object is the removal of mechanical obstruction to respiration—I mean thoracentesis, and more particularly laryngotomy and tracheotomy.

Thoracentesis is not, perhaps, very often an emergency; but laryngotomy and tracheotomy, done in cases of croup, œdema glottidis, etc., generally fail to save life, because performed too late—the patient being too much exhausted to breathe in the air for which a new entrance has been made. Artificial respiration, hypodermics of whisky and ether, cold effusions, etc., are resorted to in vain in many instances—the machinery of life cannot be set in motion again, and the cases die for want of efficient stimulation. Now, would not the intravenous injection of ammonia, in connection with artificial respiration, save many of these patients? It being proved that the treatment is without danger and followed by no bad effect, this question should not long remain unanswered.

In conclusion, I would call attention to the fact that it is not easy to perform intravenous injection through the skin. The vein collapses under the necessary pressure, and the needle is apt either to stop short and not enter the vessel at all, or to transfix it and direct the injection into the cellular tissue beyond. The only safe method to pursue is to dissect down upon the vein and expose it; the needle may then be carefully introduced until the point is felt free in the interior of the vessel.

**Treatment of Unhealthy Local and Syphilitic Sores by Immersion.**—Mr. Cooper gives the following method of treatment for phagædenic and other ulcers. The patient sits in an ordinary hip bath containing sufficient water to insure constant submersion of the affected part, for eight or ten hours a day. If the disease be in the groin, a full-sized bath in which the patient can recline may be necessary. The temperature of the water is regulated by means of a thermometer, and is kept as near 98° F. as possible by the removal and addition of small quantities of water at frequent intervals, without disturbing the position of the patient, and in winter the bath is placed near a fire. The exposed parts of the body are covered with blankets, whilst the more prominent parts are protected by air or water cushions. In the evening finely powdered iodoform or other dressing is applied and the patient goes to bed as usual. Next morning the dressing is allowed to separate in the bath; the pain attending its removal being thus avoided.

The bath is repeated day after day as long as may be necessary, general treatment according to the nature of the sore being carried on at the same time. A good purge is beneficial at the outset, followed by iron, quinine, or ammonia and bark with opium, in local sores, and by appropriate specific remedies when the patient is syphilitic. A liberal diet with plenty of milk, and little or no alcohol, answers best as a rule. Though men are here only referred to, it need hardly be said that the same treatment is equally applicable to women. The bath is to be continued for at least a day after the ulcerated surface has become quite healthy, though its use may often be protracted with excellent effect until the sore has nearly or quite healed. Some of the advantages of immersion are, its effect in nearly always quickly relieving, and often in removing entirely, the severe pain attending phagædena. Caustics are very rarely required. The ulcer is kept clean and free from discharge, without pain or trouble. When a wound has to be made by the surgeon its edges hardly ever become inoculated. The pain caused by frequent change of dressing is altogether avoided. The materials necessary for immersion being only those usually to be found in every house, it can be equally well carried out in private as in hospital practice. In the majority of cases of unhealty ulceration and sloughing, whether local or syphilitic, immersion appears to be the best and most speedy, as it is certainly the least painful of the modes of treatment hitherto adopted.—*The Lancet.*

**Glycerine as a Food.**—What is the fate of glycerine introduced into the economy? Is it decomposed or excreted? And if the latter, in what form? When large doses are given so as to produce hæmoglobinuria, the urine contains a substance which readily reduces copper, but has been said, on the ground of its effects on polarized light, not to be sugar, but to be probably a decomposition, or transformation product of glycerine.

According to Plosz, moreover, it is not capable of fermentation. It is very difficult to say whether any unaltered glycerine passes away, since the detection of a small quantity in the urine is a matter of great difficulty. It seems certain, however, that the greater part, if not all, is decomposed in the organism, and that when moderate quantities only are given, the decomposition is complete. It was observed by Weiss that the quantity of glycogen in the liver is increased by the admintstration of glycerine.

From the analogy with other substances which have a similar effect, such as albumen, gums, etc., Munk suggests that the glycerine absorbed from the intestines and carried by the portal vein to the liver is not itself transformed into glycogen, but rather, by its quick decomposition, limits the use of the liver glycogen, or furthers its formation from other materials. However this may be, the glycerine undergoes decomposition without its products having any influence on the changes in albumen, such as the carbo-hydrates exert. With reference to this, it may be remembered that glycerine has no chemical connection with the carbo-hydrates, but is rather to be regarded as an alcohol—the tertiary alcohol of the propyl series.

The solubility of glycerine renders it highly probable that the greater part of that which is taken into the stomach passes rapidly into the blood. A small part may be unabsorbed, and in the lower part of the intestine may undergo fermentation and reduction, with the formation of butyric acid, carbonic acid, etc., although this decomposition can take place only in a neutral liquid—a condition not easy to obtain in the intestine. Gorup-Besanez has also shown that in an alkaline solution, the action of oxygen in an active state breaks glycerine up into formic, propionic, and perhaps acrylic acids. There is some probability that, in the tissues, where similar conditions obtain, the same decomposition may occur; and the intermediate products, propionic and formic acids, may be further oxidized to their ultimate products, carbonic acid and water. Tcheremetjewski showed that the ingestion of glycerine causes an increase in the excretion of carbonic acid, which Catillon has affirmed may amount to seven per cent. This increase in the production of carbonic acid must be accompanied by the liberation of its equivalent of heat, and so the generation of heat should be increased by the administration of glycerine. Hence there is the highest probability that glycerine may be of service in this respect, but that it is of no value as a tissue-food.—*Lancet.*

**Therapeutics of Singultus (Hiccough).**—L. H. Washington, M.D., in the Compend of Practical Therapeutics, gives the following:

For one dose, to be repeated as necessary:

| | | | |
|---|---|---|---|
| ℞ | Comp. spts. ether............................ | 10 to 20 drops. | |
| | Cinnamon water ............................ | 10 drams. | M. |

The first dose is often all that is required.

Dr. Simon reports that a patient who had the hiccough for twenty-six hours, and been subjected to various treatment without success, was relieved almost instantaneously by inhaling three drops of amyl nitrite.

Dr. E. L. Williams writes to the Scientific Press that, "veratria powder, agitated in a vial and the stopper removed, and the dust thus raised smelled up into the nostrils, so acts upon the mucous membrane as a stimulant, as to stop the hiccough, so that the patient will not hiccough another single time after so inhaling the powder through the nose." Caution should be observed in the use of this remedy—the alkaloid veratria being highly poisonous, constial effects may be transmitted through the mucous membrane.

Dr. Oppelt mentions a case in which attacks of hiccough, "lasting from six to eight hours at a time, so that at least three-fourths of the patient's time was occupied in hiccoughing, with strength rapidly failing." After trying the usual remedies without benefit, tincture of aconite in two drop doses every three hours was given, and after the third dose hiccough ceased to a great extent, and the patient recovered under subsequent use of quinine and morphia. He was led to the use of aconite in this case, from having frequently cured sympathetic vomiting with it, after failure of everything else.

Dr. Orvil reports a case, which having resisted all the usual remedies, he injected two-thirds of a grain of pilocarpine under the skin, with the effect of producing almost instantaneous relief. In fifteen minutes the hiccough had almost entirely ceased.

A patient of Dr. Gim's, after a debauch, had hiccough for twenty hours, and was rapidly sinking. Thirty drops of chloroform given in a little sweetened water, acted like a charm; but the hiccough returning, forty drops more was given, which afforded permanent relief. An active cathartic next day set him all right.

Bromide of potassium, 40 grains, given in water every hour, sometimes quiets the nervous irritability, on which hiccough depends.

One or two doses of dilute hydrocyanic acid, one to two drops, is another good remedy.

A teaspoonful of lime water with two teaspoonfuls of milk, and a small piece of ice, given every fifteen minutes for about two hours, is excellent for irritable stomach.

In many cases exciting sudden emotion of the mind, as surprise, alarm, etc., will suddenly arrest hiccough. This acts by diverting the mind from the malady, and also as a shock to the nervous system, thus breaking up the habit.—*Ec. Med. and Surg. Journal.*

**Narcotism from Nutmeg.**—Dr. H. Barry, in St. Louis Clinical Record, says :

Mrs. N——, aged thirty-eight, mother of four children, was confined on Sabbath morning, June 29, 1879, at nine o'clock. The child was a girl, and the largest I have ever seen ; weight fourteen and one-half pounds. Labor natural and easy. Had a light spasm after the last pain. The spasm was hysterical. On the 30th the "old woman" persuaded her to take nutmeg tea. One and a half nutmegs were used in making the tea, and she drank it during the day.

About 10 p.m., she began to get drowsy. By four o'clock the next morning she was in a profound stupor. At 10 a.m. the narcotic effects of the nutmeg began to die out, and by 4 p.m. she had pretty well recovered. The symptoms were about the same as those produced by opium, and the remedies were the same.

I mention this case for the reason that nutmegs are in such general use as a condiment, that we may lose sight of their dangerous narcotic tendencies. In twenty-one years practice I have never seen such a case before, and if I had ever known that the nutmeg possessed such properties, it had completely escaped my memory, and for fear some of our numerous professional brethren may be in a like condition, I have deemed it proper to mention this case.

**Nourishment in Typhoid Fever—Unsound Logic.**—Dr. Samuel Peters in a paper on typhoid fever, in N. Y. Medical Record, refers to the importance of supporting the strength by nutriment, and adds : "I once had a case, a lady of large frame and plenty of adipose tissue, who drank every day four quarts, by measure, of pure milk. I was frequently inquired of as to the probability of her recovery. I answered that she *could not die*, because she was able to appropriate so much nourishment. She recovered," etc.

Now it so happened that the present writer also had a case of typhoid fever, a young lady of good constitution, who was able to relish and appropriate an ample quantity of the same nourishment, and who died notwithstanding. No serious symptoms occurred during the first two weeks, and no active medication was required or employed. In the third week nervous and cerebral disturbances set in, with the result mentioned. Therefore, a patient *can die* in typhoid fever, even when well nourished. Dr. Peters is not the only medical writer who has made hasty and illogical conclusions.—*Pacific Med. Journal.*

**Kava-Kava and its Blennostatic Properties.**—In a recent thesis on this subject, Dr. Dupuy states that the Kava plant contains a resin, which seems to constitute its essential therapeutic principle. The following are his conclusions concerning its medicinal properties:

1. Kava-Kava is a sialagogue.
2. Its action on the stomach is that of a bitter tonic; it improves the appetite without producing either diarrhœa or constipation, and perhaps acts as a prophylactic to catarrhal affections of the upper part of the digestive canal. Its taste is agreeable.
3. It exerts a special stimulating effect on the central nervous system; this stimulation differs essentially from alcoholic intoxication, and is called by Dr. Dupuy, *kavaic stimulation*.
4. It is not a sudorific.
5. It increases very markedly the excretion of water in the urine, and may be classed among the most efficacious of diuretics.
6. It does not produce priapism, as has been stated, but, on the contrary, it prevents it.
7. It is endowed with remarkable blennostatic properties, which manifest themselves very promptly. A chronic urethral discharge is first rendered more profuse, and is then promptly cured.
8. It is very efficacious in cases of acute urethritis or vaginitis, calming the inflammatory condition, controlling the pain during micturition, and suppressing the muco-purulent discharge from the urethro-vesical mucous membrane.

These results are probably due to the combined diuretic and blennostatic actions of the drug.

The anti-catarrhal action seems to be due to the resin, and the diuretic effects to a neutral, crystallizable principle called kavaine, and perhaps also to an alkaloid not yet discovered, whose presence would explain more satisfactorily the phenomena excited in the central nervous system, as well as the alterations in the circulation and secretions of the uro-genital apparatus. It possesses over other blennostatic agents, according to Dr. Dupuy, marked advantages, inasmuch as it produces neither diarrhœa nor constipation, is taken with pleasure, increases the appetite, relieves or controls entirely the pain during micturition, changes completely the nature of the discharge, and produces a cure in a very short space of time—ten days.—*La Tribune Medicale.*

**A Novel and Successful Remedy for Hay Fever.**—Dr. C. M. Sebastian, of Martin, Tenn., in a paper read before the Southwestern Kentucky Medical Association, has the following in regard to this disease:

"After patient study I was convinced that if the trouble was intrinsic to the system there would not be this uniform relation in the spring time. I was therefore led to search for the specific materia morbi among the conditions incident to spring. Numerous substances, such as ipecac, ammonia, carpet dust, etc., when in a state of atomic suspension in the air, to certain persons of peculiar susceptibilities become irritants, capable of producing asthma.

"Reasoning by analogy, I concluded the active agent in this case must either be pollen wafted from a thousand fragrant flowers; or, perhaps, bearded spores torn from the green velvet surface of the half-fledged foliage. Dogmatically assuming that the one or other of the two latter propositions was inevitably correct, I cast about for the means of keeping the morbific principle extraneous of the system. I at last, with the view of accomplishing this end, prescribed to my patient the constant wearing of a lady's thick veil. I told him that since the measure was novel and untried, it would subject him to the annoyance of being made ridiculously conspicuous, but that he must bear all in order to prevent the coming trouble. So next morning a figure, closely veiled, appeared like a spectre on our thoroughfares. Who is that? What does that mean? was heard on every hand, but the mysterious apparition moved grimly and silently on. Some thought it was Banquo's ghost, and others that it was an escaped lunatic. But like Ajax defying the lightning, he bravely stemmed the current of the storm and remained faithful to his remedy, which in due time demonstrated itself to be the sovereign balm to his ills.

"The flowers shed their fragrance, the birds trilled their songs, and the golden days of spring chased each other down into the province of summer, and yet the disease came not. The next spring, and the next, which is the season just now about to pass out, Mr. Parker again resorted to his remedy, with the same happy effect, and to-day, there is not a healthier, happier old man in the classic but ill-starred town of Martin, Tenn., than Mr. Parker.—*Med. Herald.*

**Foul Water and Dysentery.**—We have for some years been accustomed to connect the continual occurrence of dysentery, chiefly in India and China, with the imbibition of bad water, or water fouled by excreta or other unhealthy material. An interesting additional proof of this connection is recorded in the Indian Medical Gazette, from the pen of Dr. Payne, superintendent of asylums in the Calcutta Presidency, who, in his report for 1876, remarked emphatically on the exemption from dysentery that the inmates of the lunatic asylum at Calcutta had enjoyed during the previous year. It appears that the resident surgeon, like his predecessors, had been much exercised by the continued prevalence of this disease in the institution, and had adopted every possible means for its eradication. It seems, however, that a large proportion of the patients had for some time been affected with round intestinal worms. The medical officer thus explains the history of the epidemic, and the means that were adopted for its removal:

"Outside the latrines there were shallow masonry reservoirs of water, in which the people resorted for ablution after using the latrines. Early in the year, in the course of a morning visit, I noticed that a lunatic, after washing himself, lifted a handful of the water to his mouth, and there appeared at once an explanation of the great quantity of worms in the asylum; for it was clear that when any one discharged the ova they would readily find their way to the stomachs of others adopting this practice, and it was equally clear that, so long as access were allowed to stagnant water for ablution, the practice would continue. A plan was devised by which this use of water should be made impossible. The reservoir was broken up. A pipe was provided, supplied with water, and taps fitted to its side at intervals. On opening either of the taps a stream of water projected horizontally and fell on a platform which sloped to a drain. The lunatic seated on the platform, opposite the stream, or rather in it, could wash freely, but be unable to make any further use of water which had once touched his body. This appeared to be entirely successful, for after a short time no more was heard of round worms in the asylum."

But a still more satisfactory sequel occurred in the cessation, or, at all events, remarkable diminution in cases of dysentery, which dropped from 103 in 1875 to 35 in 1876, and it is remarked, "from 1876 to the present time the asylum has remained free from both worms and dysentery, though the last two years have been periods of excessive mortality in the neighborhood." We have not space to follow the writer through the details of many painstaking observations, in which he shows, and, as we think, quite conclusively, that no other causes, direct or indirect, were at work to aid, even to a remote extent, in producing this result.

The fact is a valuable contribution to the proof of a distinct connection between foul water and dysentery. This disease, in its chronic form, always furnishes a large contingent of patients to the *Dreadnought* Hospital at Greenwich; but it has been observed by the officers of that institution that, as more care has been taken recently in supplying water to ships at Calcutta. Hong Kong, etc., so the severity of the cases sent home has much diminished. The substitution of iron tanks for casks was a very important step in the right direction, and most good ships now cease to take their supply of drinking-water from the rivers in India and China.—*Lancet.*

**Influence of Syphilis on the Cure of Wounds.**—We translate from *Les Annales de Dermatologie et de Syphiliographte* the conclusions of a remarkable work of Dr. Dudterhoff.

Wounds or sores exposed to a continual irritation may, during the period of syphilitic contagion, be the seat of syphilitic efflorescences without retarding the cure.

Severe wounds favor the latent stage of syphilis; but when the wound is well or almost so, syphilis may appear at the place of traumatism or any other part of the body.

Latent syphilis does not generally interfere with the cure of wounds by first intention after surgical operations.

Syphilis of the bones predisposes to their fracture and is an obstacle to their union; although a specific treatment does not prevent consolidation of fractures; but in some cases that consolidation is prevented by syphilitic cachexy or active mercurial treatment.

In cases of inveterate syphilis, especially on weak patients, wounds and sores are sometimes subject to gangrene, which can be cured by an antisyphilitic treatment.

It is not proven that constitutional syphilis predisposes wounds to hemorrhage; it does not favor either the appearance of pyemia.

**Transplantation of a Dog's Cornea to the Human Eye.**—Mr. Schœler, in *La Revue Medicale,* relates the case of a man, aged twenty, one of whose eyes was atrophied, while the other had just lost the entire cornea through its prolonged ulceration. The iris, covered with granulations, was laid entirely bare, the lens had dropped out. The patient had merely luminous sensations. Mr. Schœler operated by cutting a large upper conjunctival flap, capable of covering the whole extent of the cornea; then below, a small flap intended to be united by points of suture to the upper flap that was turned down, the epithelial surface of both flaps being turned back against the surface of the globe. By means of a trephine he removed from the eye of a chloroformed dog a circular portion of the cornea, about 9½ millimetres in diameter. This cornea being applied to the vacant place in the human eye, he brought down in front of it the large conjunctival flap, which he united by catgut sutures to the small flap. The transplanted cornea was thus held in position and protected by the conjunctival flaps. At the end of three days the sutures fell out, the conjunctival flap was adhering to the transplanted cornea, and the latter to the margin of the sclerotic. There was an anterior chamber visible where the conjunctiva was deficient. But on the following days the cornea gave trouble, and finally became of a milky tint—an ulcer appeared. By degrees vessels found their ways into the periphery of the cornea and reached its centre. After the sixth week the conjunctival flap was detached. Eight days afterward the cornea was flat, very opaque in the center, but translucid at the periphery so as to let the iris be seen. The vision is, however, very slight; the movement of the hand can be distinguished at a distance of half a foot from the eye.—*Half-Yearly Comp.*

**The Ethylate of Sodium.**—Dr. Purdon, in London Lancet, says:

I have quite recently been trying the ethylate of sodium at the Belfast Skin Hospital, and beg to make the following very brief remarks.

My first case was that of a nævus, situated on the forehead, communicating through the bone, with cerebral circulation. The patient, a boy, aged three years; the nævus about the size of a small walnut. Four applications of the ethylate of sodium removed the deformity. I think Dr. Richardson is right when he says that this remedy is a specific for nævus. I have also removed a small patch of cutaneous cancer on the lower lip by the ethylate; whilst three cases of lupus and one of warty growth on the back of the hand are progressing satisfactorily under treatment. The four results obtained by the application of the ethylate of sodium—1st, removal or absorption of water from the tissue into the ethylate; 2d, destructive action of a caustic from the caustic soda that is formed; 3d, coagulation from the alcohol that is produced; and 4th, prevention of decomposition of the dead organic substance that is formed—were observed in the case of nævus.

I merely jot down the above for the purpose of calling attention to a remedy that is likely to admit of a wide range of action.

**Cause of Death in Uterine Injections.**—Dr. J. K. Chadwick gives the following summary of causes of death from uterine injections:

The manner and suddenness of the death can be explained on one only of three theories: that the woman died, first, of shock; second, of embolism; third, of the entrance of a large volume of air into the blood-vessels.

1. Although authorities state that death from shock may result from uterine and vaginal injections, yet no reported cases are known to witness in which this is clearly made out; hence we are led to believe that the term shock, which is now used in many cases where no demonstrable cause of death is found, at the autopsy, has been applied in instances in which the most scientific pathology of the present day would show to have been due to embolism or air in the veins.

2. Embolism. Death from this cause is very rarely instantaneous. If it occurs, it must result from an arrest of a clot at or near the heart, and would be disclosed by careful autopsy. Besides, days would be required to allow any coagula that might be found in the uterine veins to become so disintegrated as to be taken up by the blood and carried forward toward the heart.

3. The entrance of air into the blood-vessels has been shown to have occurred in very many instances, and to have caused instant death. Many theories have been advanced to account for this result. I believe this to be the true explanation . The regurgitation of the blood in the large arteries, which takes place as soon as the impulse of the heart's contraction is removed, is competent to *close* the valves at the orifice of such vessels. Now if air is substituted for blood, the valves do not close, and the circulation of the blood is arrested, however vigorously the heart may continue to beat. Instant death is the result. Further, in this instance the detachment of a portion of the placenta opened some of the uterine sinuses, and the autopsy showed that they were not plugged up by coagula.—*London Med. News.*

**The Contagium of Syphilis.**—Numerous experiments with the contagium of syphilis have recently been made by Klebs, and are summarized by the London Medical Record. Man and the lower animals have been inoculated, and clinical and microscopical observations carefully made. The results of the experiments are summed up in the following conclusions :

1. Syphilis in man can be communicated to lower animals by inoculating them with portions of the syphilitic new formations. But the course of the disease is not the same for each genus. With apes the disease is quite the same as in man; rabbits have given other results, which, if not so striking, nevertheless will not allow any mistake as to their similarity with the disease in man.

2. In syphilitic new formations in man, certain low fungoid organisms are found, which develop into peculiar forms—helicomonads.

3. By the transference of these to selected animals, changes are set going which correspond not only with those of genuine syphilis in man, but also with those of inoculated syphilis of animals.—*N. Y. Med. Journal.*

**Honor to Dr. Crawford W. Long.**—Mr. F. B. Carpenter, of New Xork, has just finished a nearly full-length portrait of Dr. Crawford W. Long, late of Athens, Ga., at the order of the alumni of the Georgia University. It is in honor of the discovery made by Dr. Long, March 30, 1842, that inhalation of sulphuric ether renders a patient insensible to the pain attending a surgical operation. It will be received and presented to the Georgia Legislature by United States Senator Gordon.—*Ex.*

**Coffee in Cholera.**—It is asserted that strong coffee, without sugar or milk, given in teaspoonful doses every ten minutes, will arrest the vomiting of cholera infantum; and that a tablespoonful given frequently to adults will relieve the vomiting of cholera morbus. It is an article which has the merit of being without danger at all events.

**Chronic Bright's Disease.**—Dr. N. L. Guice, in a communication to the American Bi-weekly, gives the history of a case of chronic Bright's disease apparently cured by the use of iodide of potassium. The patient, aged 50, had suffered from malaria, but no history of syphilis was given. He became affected with Bright's disease, with all the characteristic bad symptoms. His urine contained a large amount of albumen, together with granular and hyaline casts. Iodide of potash was ordered in doses of gr. v. three times a day, and this was gradually increased to gr. xij. during the course of treatment. The symptoms slowly improved, and by the end of six months the patient appeared in every respect well. Iron and bitter tonics supplemented the potash. Five other cases of the successful use of the iodide in this disease have been reported.—*N. Y. Med. Record.*

**Poisoning by Rhus Radicans.**—J. M. Ward, M.D., wishes to call the attention of the profession to the use of the liq. chlo. soda, or Labarraque's solution, in all cases of rhus poisoning. The acid poison requires an alkaline antidote, and this solution meets the indication fully. When the skin is unbroken it may be used clear three or four times a day, or in other cases diluted with from three to six parts of water. After giving this remedy a trial no one will be disposed to try anything else. It is one of the most valuable external agents known to the profession, and yet seldom appreciated, and but rarely employed. It will sustain its reputation as a local application in erysipelas, burns, and scalds.—*N. Y. Med. Record.*

**Contagiousness of Abdominal Typhus.**—Abdominal typhus was brought into the lunatic asylum of Werneck by an insane patient. The hygienic conditions of the institute are excellent. The drinking water, cloaca, cleanliness of the grounds, and all the conditions of the place are unfavorable to the propagation of the disease. Many persons were infected by the soiled clothes, and most of the attendants and patients who slept near the case above referred to were also attacked. When a patient with typhus was carried into another apartment where the disease had not existed, the persons near the former were soon attacked. Dr. Schwabb concludes from this that abdominal typhus is transmissible from person to person.—*N. Y. Med. Journal.*

**Lemon Juice for Hypertrophied Tonsils.**—Saint Germain has found lemon-juice a very simple and efficacious remedy for the suppression of hypertrophied tonsils. In young subjects, he pencils the tonsils with lemon-juice twice a day. A cure is usually obtained in two weeks. He does not consider more heroic treatment justifiable till this remedy has failed.—*Revue de Therapeutique.*

**To Hasten the Action of Quinine.**—Dr. Starke (Berliner Klin-Wochenschrift) advises that before swallowing powders or pills of quinia, a weak tartaric acid lemonade be taken. This procedure not only accelerates the solution and absorption of the quinia, rendering its physiological action much more prompt, but also obviates that unpleasant gastric irritability so common after the administration of large doses of this drug.—*Cin. Lancet and Clinic.*

